# Capitalizing on the Teachable Moment: Osteoarthritis Physical Activity and Exercise Net for Improving Physical Activity in Early Knee Osteoarthritis

**DOI:** 10.2196/resprot.2553

**Published:** 2013-05-09

**Authors:** Linda C Li, Sydney Lineker, Jolanda Cibere, Valorie A Crooks, Catherine A Jones, Jacek A Kopec, Scott A Lear, James Pencharz, Ryan E Rhodes, John M Esdaile

**Affiliations:** ^1^Arthritis Research Centre of CanadaRichmond, BCCanada; ^2^The Arthritis Society, Ontario DivisionToronto, ONCanada; ^3^Division of RheumatologyUniversity of British ColumbiaVancouver, BCCanada; ^4^Department of GeographySimon Fraser UniversitySurrey, BCCanada; ^5^Faculty of Rehabilitation MedicineUniversity of AlbertaEdmonton, ABCanada; ^6^School of Population and Public HealthUniversity of British ColumbiaVancouver, BCCanada; ^7^Faculty of Health SciencesSimon Fraser UniversityVancouver, BCCanada; ^8^Credit Valley HospitalMississauga, ONCanada; ^9^Department of Exercise Science, Physical and Health EducationUniversity of VictoriaVictoria, BCCanada

**Keywords:** osteoarthritis physical activity, Internet, lifestyle intervention, theory of planned behavior

## Abstract

**Background:**

Practice guidelines emphasize the use of exercise and weight reduction as the first line of management for knee osteoarthritis (OA). However, less than half of the people with mild OA participate in moderate intensity physical activity. Given that physical activities have been shown to reduce pain, improve quality of life, and have the potential to reduce the progression of joint damage, many people with OA are missing the benefits of this inexpensive intervention.

**Objective:**

The objectives of this study are (1) to develop a behavioral theory-informed Internet intervention called Osteoarthritis Physical Activity & Exercise Net (OPEN) for people with previously undiagnosed knee OA, and (2) to assess the efficacy of the OPEN website for improving physical activity participation through a proof-of-concept study.

**Methods:**

OPEN was developed based on the theory of planned behavior. Efficacy of this online intervention is being assessed by an ongoing proof-of-concept, single-blind randomized controlled trial in British Columbia, Canada. We are currently recruiting participants and plan to recruit a total of 252 sedentary people with previously undiagnosed knee OA using a set of validated criteria. Half of the participants will be randomized to use OPEN and receive an OA education pamphlet. The other half only will receive the pamphlet. Participants will complete an online questionnaire at baseline, 3 months, and 6 months about their participation in physical activities, health-related quality of life, and motivational outcomes. In addition, we will perform an aerobic fitness test in a sub-sample of participants (n=20 per study arm). In the primary analysis, we will use logistic regression to compare the proportion of participants reporting being physically active at or above the recommended level in the 2 groups, adjusting for baseline measurement, age, and sex.

**Results:**

This study evaluates a theory-informed behavioral intervention at a time when people affected with OA tend to be more motivated to adopt an active lifestyle (ie, at the early stage of OA). Our approach, which consisted of the identification of early knee OA followed immediately by an online intervention that directly targets physical inactivity, can be easily implemented across communities.

**Conclusions:**

Our online intervention directly targets physical inactivity at a time when the joint damage tends to be mild. If OPEN is found to be effective in changing long-term physical activity behaviors, it opens further opportunities to promote early diagnosis and to implement lifestyle interventions.

**Trial Registration:**

Clinicaltrial.gov: NCT01608282; http://clinicaltrials.gov/ct2/show/NCT01608282 (Archived by WebCite at http://www.webcitation.org/6G7sBBayI)

## Introduction

### Background

Arthritis is the most common cause of severe chronic pain and disability [1,2], affecting about 4.6 million Canadians (aged 15 or older) and projected to affect 7 million by 2031 [2]. It is estimated that the majority of these people are affected by osteoarthritis (OA) [3]. Being physically active has been shown to reduce pain, improve quality of life [4-6], and have the potential to slow the progression of joint damage [7]. However, the gap between the *knowledge* about OA management and the *action* of being physically active is extremely large.

Sedentary lifestyle and obesity are predictors of poor health outcomes in people with OA [8-10]. Recent guidelines by the OA Research Society International (OARSI) specifically recommend the use of aerobic, muscle strengthening, and water-based exercises, as well as weight reduction as first line management of knee OA [11], but the majority of people with OA are physically inactive. In 2002, the Arthritis Foundation developed 22 indicators to assess the quality of care in OA [12-14]. When the indicators were applied to a community-based sample in Ontario, 40.1% of people with OA who had no contraindication to exercise had tried exercise [15]. A survey of 1713 people with OA in British Columbia, Canada found that although 79% reported spending time “walking for exercise in the past week”, the majority walked less than one hour per week [16].

Several factors are associated with low participation in physical activities in people with arthritis, some of which are related to the disease (eg, higher levels of pain and fatigue [17-19]), sociodemographics (eg, lower education [20] and income [21]), the person (eg, other commitments, lack of time and motivation [17], doubts about the effectiveness of exercise [19]), and other enabling factors (eg, access to transportation [18], weather [17,18]). People who are newly diagnosed with knee OA tend to have mild pain, stiffness, and functional disability [22]. For these patients, disease-related factors may be a less important barrier to engaging in physical activities. It should be noted that factors associated with physical activity participation were often studied without an overall explanatory framework, making it challenging to develop interventions that work equally well for people with different needs.

### Adapting Knowledge to the User’s Context: Theory-Informed Lifestyle Interventions

In health promotion, the Theory of Planned Behavior (TPB) has been used extensively to understand and predict health-related/lifestyle change behavior [23]. TPB posits that the adoption of a health *behavior* is driven by the person’s *intention* and *perceived behavioral control (PBC)* [24]. The latter represents the perceived skills/ability, resources, and opportunities of performing the behavior [25]. Furthermore, the strength of *intention* is determined by PBC, the *attitudes* toward the behavior (ie, affective attitude—enjoyment, pleasure evaluations about the behavior; and instrumental attitude—benefit, utility evaluations about performing the behavior) and *subjective norm* (eg, the perception of how others view the behavior and the importance of these views to the person). In a metaanalysis, Hagger et al [26] reported that intention and PBC accounted for about 30% of the variance in physical activity *behaviors*, while attitude and PBC accounted for about 40% of the variance in *intention*. A 2009 metaanalysis by Rhodes found that interventions targeting affective judgement constructs such as affective attitude (eg, enjoyment, pleasure) were effective for predicting intention and physical activity behaviors above the instrumental attitude construct [27].

Although the TPB has not yet been used in the study of physical activity in people with early OA, it has been applied in comparable populations, including older adults [28], those with painful intermittent claudication [29], and those who are obese [30], to inform the development of interventions for improving physical activity. For example, Godin observed that in people with a body mass index (BMI) ≥30, PBC, past behavior, and anticipated regret (ie, the perceived feeling of regret if the behavior is not performed) substantially improved the predictive power of intention, explaining 59% of the variance [30]. Another study examining social cognitive constructs in people awaiting joint replacement surgery for end-stage OA found that pain was a predictor of pre-operative physical activity [31]. Hence, proper monitoring and control of symptoms may be important for people with knee OA during physical activity.

There is no consensus on the timing to offer physical activity interventions, but sociopsychological research suggested that after a major life experience (eg, having a child) or a health event (eg, a new diagnosis), people tend to be more amenable to adopting healthy behaviors [32,33]. This “teachable moment” [34] is thought to be the ideal time for lifestyle interventions because people are more motivated. Systematic reviews of chronic diseases such as cancer suggest that people who are newly diagnosed are more likely to respond to interventions aimed at smoking cessation and healthy eating [34-36]. A recent study in people with previously undiagnosed knee OA also found that about 40% started exercising within the first month after receiving a pamphlet on OA and completing a volunteer-led self-management program [37]. Although, the mechanism of this behavior change was unclear, the diagnosis of OA appeared to present a “teachable moment” for engaging people who have been sedentary to become physically active.

### Why Use Internet-Based Physical Activity Interventions?

In recent years, computerized programs have gained popularity as tools for promoting healthy lifestyles because of their potential to improve the delivery and presentation of information. Evidence suggested that computerized health information programs could improve disease knowledge and clinical outcomes in people with rheumatoid arthritis, diabetes, asthma, and hypertension [38], as well as self-care behaviors and patient satisfaction in those with chronic conditions [39]. A 2008 systematic review suggested that mediated interventions such as telephone prompts, emails, and websites can increase duration of walking in healthy participants, with the added advantage of saving time for busy individuals because none of these interventions require a visit to an exercise professional [40]. They also have the benefit of reaching patients outside of urban centers who are often marginalized with respect to health care.

To capitalize on this “teachable moment”, our goal was to develop a Web-based tool, Osteoarthritis Physical Activity and Exercise Net (OPEN), and evaluate its ability to improve physical activity participation in people with early knee OA. OPEN has interactive modules that allow users to prioritize their daily activities, set goals, and find venues where they can participate in different types of activities according to their preferences and the local availability. We have begun the participant recruitment phase of this study, with the following objectives and goals:

1. To develop a behavioral theory-informed Internet intervention for people with early knee OA.
2. To assess the efficacy of OPEN through a proof-of-concept randomized controlled trial (RCT). The *primary goal* of this ongoing RCT was to determine if OPEN could increase participation in physical activity in people with previously undiagnosed, early knee OA at 6 months. We hypothesized that the Internet intervention plus an information pamphlet about OA would improve participation in physical activities in persons with early OA, compared to those who would receive only the pamphlet (ie, controls). Our *secondary goals* were to assess: (1) whether the intervention has an effect on knee pain, stiffness, and physical function at 6 months, (2) whether differences between groups could be explained through a mediation model based on a behavioral theory (ie, the TPB), and (3) in a subsample of participants, the agreement between self-reported exercise behaviors and a performance-based measure of physical activity.

Guided by Graham’s Knowledge-to-Action process [41], this study will directly address a key recommendation from the 2005 Summit on Standards for Arthritis Prevention and Care:

Every Canadian must be informed about the importance of achieving and maintaining a healthy body weight, and actively encouraged to engage in physical activity to prevent the onset and worsening of arthritis. [42]

## Methods

### Overview

Our research plan was guided by the 8-phase Action Cycle of Graham’s Knowledge-to-Action Process [41]. Co-developed with the Centre for Digital Media and hosted by the Arthritis Research Centre (ARC) of Canada, OPEN was designed to mainly target *perceived behavioral control* (ie, the person’s skills/knowledge, resources, and opportunities to be physically active) and *affective attitude* (ie, enjoyment)[27]. The website consists of 4 main components: (1) information on OA, benefits of physical activity in OA, and the association between sedentary lifestyle and poor outcomes (to target knowledge), (2) tips about how to be physically active (to target skills and opportunities), (3) an interactive calendar for goal setting (to target resources), (4) local resources (walking trails, parks, shopping malls for indoor walking) and community fitness facilities using Google Maps (to target resources and enjoyment).

To populate OPEN with these local resources, the team (researchers, patient/consumer, and health professional collaborators) first defined the breadth of resources to be included. Next, the geographic locations of these resources were determined (through Web searches and strategic calling) for all BC communities with populations of 5000 or greater and geocoded (ie, a postal code or street address was recorded). Following this, local resources were compiled onto a Google Map that was embedded within the OPEN website. Tags were added to each resource to provide information such as operating hours and contact information.

### Randomized Controlled Trial

The efficacy of OPEN is currently being assessed by a proof-of-concept, single-blinded RCT. Individuals with early OA will be identified using validated criteria developed by Marra at al [22]. Eligible participants are those who:

1. have had pain/discomfort in or around the knee during the previous year lasting over 28 separate or consecutive days,
2. are age 50 years or older,
3. have no previous physician diagnosis of OA, rheumatoid arthritis, psoriatic arthritis, gout, ankylosing spondylitis, polymyalgia rheumatica, connective tissue diseases, or fibromyalgia,
4. have no history of using disease-modifying anti-rheumatic drugs or gout medications,
5. have no prior knee arthroplasty,
6. have not had knee surgery within 4 months prior to enrolling in the study,
7. have no history of acute injury to the knee in the past 6 months,
8. have been physically inactive (defined as participation in moderate intensity activities less than 150 minutes a week) within 6 months prior to study,
9. are not using medication that may impair physical activity tolerance (eg, beta blockers), and
10. have Internet access and used their email accounts.

People who may be at risk by exercising, as identified by the Physical Activity Readiness Questionnaire (PAR-Q) [43], will be asked to obtain permission from their physicians before enrolling in the study.

Individuals will be mainly recruited from the following sources: (1) community health centers across Metro Vancouver, (2) the ARC website, newsletters, and Facebook site, (3) social networking websites (eg, Craigslist, Kijiji), (4) local television network (community event posting), and (5) local newspapers.

The research coordinator will contact eligible individuals and obtain consent via password-protected email documents. After completing the baseline measures, they will be randomly assigned to the Internet intervention group or the control group in 1:1 allocation ratio. Randomization will be performed using computer-generated random numbers in unequal blocks, which is necessary to ensure adequate allocation concealment.

The intervention group will receive an emailed password. Participants will receive an automatically generated email prompt to access the website every 2 weeks, with a short newsletter about the ongoing projects at ARC, for the first 3 months. The website will remain accessible throughout the study, but no further prompting emails will be sent after 3 months. In addition, they will receive, by email, an education pamphlet. It will contain information about OA, physical activity, and other treatments.

The control group will receive the same pamphlet by email. For the first 3 months, participants will also receive the same newsletter about the ongoing projects at ARC every 2 weeks by email. During the intervention period, both groups will be able to contact a physical therapist for a consultation via email if they experience increased discomfort after activities.

Outcome measures will be administered online at baseline, 3 months, and 6 months. The primary outcome will be the proportion of participants to meet the American College of Sports Medicine physical activity recommendation of 150 minutes or more of weekly physical activity (moderate or heavy intensity) at 6 months, as measured using the modified Minnesota Leisure Time Physical Activity Questionnaire (MLTPAQ). The MLTPAQ assesses the frequency and amount of time spent on 63 activities in 8 categories: walking, conditioning exercise, water activities, winter activities, sports, garden activities, home repair activities, fishing, and hunting. The average time spent on moderate and heavy physical activity and average weekly energy expenditure (kilocalories/week) will be calculated using the standardized intensity code associated with each activity [44]. It has shown test-retest reliability in both men and women (*r*=.79-.82) [45] and has been validated against caloric intake and treadmill tests [46-48].

Secondary outcomes will be measured with the Knee Injury & OA Outcome Score (KOOS) [49,50]. The KOOS consists of 5 subscales: knee pain, stiffness, daily activity, sports/recreation, and quality of life. It was originally developed for people recovering from injuries such as anterior cruciate ligament and meniscus injury, and has been validated in people with OA [49,50]. KOOS includes all items of the Western Ontario MacMaster OA Index (WOMAC) in its original format [51] and has a normalized aggregate score ranging from 0 (worst outcome) to 100 (best outcome). Motivation for physical activity will be measured with Rhodes’s 7-point Likert-type TPB questionnaire [52-54]. It consists of 16 items measuring all components of the TPB model, including behavioral, normative, and control beliefs. Previous studies using this measure have shown good predictive validity and internal consistency in adult populations [52-54]. Demographic variables and comorbid conditions will also be collected at baseline. Website statistics (frequency and duration of use, intervention group only) and adverse events (falls, cardiovascular, and musculoskeletal events) [55] will be tracked monthly.

Finally, in a convenient subsample of participants (n=20 per arm), we will perform an aerobic fitness test at baseline, 3 months, and 6 months. Aerobic fitness tests (VO_2Peak_) will be conducted by an assessor who is blinded to the group assignment. Heart rate, blood pressure, and VO_2_ will be recorded at rest, during each exercise stage, and in recovery.

### Sample Size and Data Analysis

Based on the Pharmacist Identification of New, Diagnostically Confirmed OA (PhIND-OA) study [22,37], 40% of residents from British Columbia in Canada (N=194, mean age=62 years) started to exercise after receiving an OA diagnosis and minimal intervention (ie, a pamphlet and the self-management program). We expect that, if OPEN is efficacious, 60% of the intervention group will meet or exceed the recommended level of physical activity at follow-up. Taking into account a 15% loss to attrition over 6 months, 80% power to detect a difference of 20% in physical activity rates between groups and alpha level of .05, a total of 252 participants (126 per group) will be needed. To validate the self-reported physical activity measure, the MLTPAQ, a subsample of 40 participants will be recruited. Assuming we observe a Pearson correlation of .5 for MLTPAQ and VO_2Peak_, a 95% confidence interval around the estimate would range from 0.26 to 0.74.

As the aim of a proof-of-concept study is to demonstrate evidence of efficacy, a per-protocol analysis will be performed. For the primary outcome, dichotomized physical activity participation (ie, yes/no to meeting the American College of Sports Medicine recommendation) from baseline to 3 and 6 months will be analyzed in a logistic regression model after adjusting for the baseline measurement, age, and sex. Intention-to-treat analysis will assess the robustness of the findings. Analysis of covariance (ANCOVA) will analyze the difference in KOOS scores (overall and subscales) between the groups over time. No adjustment will be made for multiple comparisons because Type II error is a greater concern than Type I error in proof-of-concept studies [56,57].

We will examine the mechanisms of OPEN on physical activity participation by conducting mediation analysis using the bootstrapped sampling distribution model by Preacher and Hayes [58,59]. Changes in TPB variables over the intervention period (ie, between baseline and 3 months) will be examined as potential mediators on physical activity behavior at 3 months. In addition, changes in TPB variables over the entire study period (ie, baseline to 6 months) will be examined as potential mediators on physical activity participation at 6 months. Finally, an exploratory analysis using Pearson’s correlations will examine the association between MLTPAQ (ie, energy expenditure) and the VO_2Peak_.

### Timeline

Development and usability testing of OPEN were completed in November 2012 and RCT recruitment commenced in December 2012. Final follow-up assessment is expected to conclude in January 2014. The study protocol has been approved by the University of British Columbia Clinical Research Ethics Board (certificate number: H12-00493).

## Discussion

The proposed study will be one of the first to evaluate a theory-informed behavioral intervention at a time when people tend to be more motivated to adopt an active lifestyle (ie, at the “teachable moment”). Our approach, consisting of identifying people with early knee OA using a set of validated criteria [22] followed immediately by an online intervention that directly targets physical inactivity, can be easily implemented across communities. This proof-of-concept study will provide a foundation to further study and implement lifestyle interventions in managing chronic musculoskeletal conditions. If the intervention is found to be effective in changing physical activity behaviors, it will open further opportunities to promote early diagnosis and to implement lifestyle interventions. Conversely, if the intervention shows no difference in improving physical activity behavior compared to the control, this study will still offer the opportunity to examine relationships between the TPB constructs and physical activity participation.

The OPEN project also has potential to improve primary and community-based care in people with arthritis. The value of this project is summed up by our Knowledge User Co-Investigator and a primary care physician, Dr. James Pencharz:

I see the impact of physical inactivity on my patients daily. Even though I work within an interdisciplinary team environment specifically designed to manage chronic disease, we still struggle to consistently motivate and educate our patients about how to increase their physical activity…I see the innovative approach of OPEN as an excellent initiative to educate patients about osteoarthritis, but more importantly customize and realize their physical activity goals. Simply, we need this type of tool in our clinical practice.

The partnership with Centre for Digital Media allows for the development of OPEN using the latest digital media technologies and provides training to digital media students in health research. Once the research is completed, OPEN will be available free of charge for public use.

## Abbreviations

ANCOVA: analysis of covariance
ARC: Arthritis Research Centre of Canada
BMI: body mass index
KOOS: Knee Injury & Osteoarthritis Outcome Score
MLTPAQ: Minnesota Leisure Time Physical Activity Questionnaire
OA: osteoarthritis
OARSI: Osteoarthritis Research Society International
OPEN: Osteoarthritis Physical Activity and Exercise Net
PAR-Q: Physical Activity Readiness Questionnaire
PBC: perceived behavioral control
PhIND Study: Pharmacist Identification of New, Diagnostically Confirmed OA Study
RCT: randomized controlled trial
TPB: Theory of Planned Behavior
VO_2Peak_: maximum aerobic capacity
WOMAC: Western Ontario MacMaster OA Index
